# Autocrine Sonic hedgehog signaling promotes gastric cancer proliferation through induction of phospholipase Cγ1 and the ERK1/2 pathway

**DOI:** 10.1186/s13046-016-0336-9

**Published:** 2016-04-02

**Authors:** Zhai Ertao, Chen Jianhui, Chen Chuangqi, Qin Changjiang, Chen Sile, He Yulong, Wu Hui, Cai Shirong

**Affiliations:** Department of Gastrointestinal Surgery, The First Affiliated Hospital of Sun Yat-sen University, 58 Zhongshan 2nd Road, Guangzhou, Guangdong 510080 China

**Keywords:** Gastric cancer, Autocrine, Sonic hedgehog, Proliferation, Prognosis

## Abstract

**Background:**

Sonic hedgehog (SHH) plays critical roles in cell growth and development. Tumor cells express SHH, which can promote cell proliferation and epithelial-to-mesenchymal transition. However, the autocrine SHH pathway has not been described in gastric cancer. The aim of this study was to explore molecular mechanisms underlying autocrine SHH signaling in gastric cancer cells.

**Methods:**

SHH expression was assessed using immunohistochemistry and the results were compared with clinicopathologic parameters, including survival. Using gastric cancer cell lines, we measured SHH mRNA and protein expression, and studied the effects of SHH signaling on cell proliferation and SHH secretion. We also studied the effects of an inhibitor of PLC-γ1 on phosphorylation of phospholipase Cγ1 and extracellular signal-regulated kinases (ERK)1/2.

**Results:**

SHH protein expression in gastric cancer tissue was significantly higher compared with that in normal gastric tissue (*P* < 0.001), and the increased expression was significantly associated with pT staging (*P* = 0.004), pN staging (*P* = 0.018), pM staging (*P* = 0.006), and pTNM staging (*P* < 0.001). In multivariate analyses, overall survival in gastric cancer was significantly shorter in cases with high SHH expression (HR = 1.734, 95 % CI: 1.109–2.713, *P* = 0.016). The AGS and SGC-7901 gastric cancer cell lines expressed SHH mRNA and protein. In these cell lines, SHH promoted carcinogenesis through activation of the PLCγ1-ERK1/2 pathway, resulting in increased cell proliferation and survival.

**Conclusions:**

Increased SHH expression is associated with shorter survival in gastric cancer patients, and SHH could represent a useful biomarker or therapeutic target for this disease.

## Background

Gastric cancer (GC) is the fifth most common form of carcinoma and the second leading cause of cancer-related mortality worldwide [1]. It is estimated that there are approximately 400,000 new cases in China annually, comprising approximately 43 % of the global total [2]. Despite advances in chemotherapy and surgery, the prognosis of patients with advanced GC remains poor. For instance, the 5-year survival rate is only 4 % for stage IV GC [3]. Targeted molecular therapy provides greater specificity and selectivity than traditional treatments, decreasing the likelihood of drug resistance and chemotherapy-associated side effects [4]. Despite their superior efficacy against many tumors, the effects of targeted drugs on GC remain unsatisfactory. Therefore, it is necessary to further understand the molecular mechanism underlying GC development and to design new targeted drugs for improving the prognosis and treatment of GC patients.

The Hedgehog signaling pathway plays a critical role in stem cell maintenance and the specification of patterns related to cell growth and differentiation during embryonic development [5–7]. Hedgehog was first identified as a secreted protein in Drosophila melanogaster [8, 9]. Hedgehog has three mammalian counterparts: Sonic hedgehog (SHH), Indian hedgehog, and Desert hedgehog. The SHH signaling pathway is activated by SHH binding to the Patched (Ptch)-Smoothened (Smo) membrane-receptor complex. Upon activation in vertebrates, Smo promotes nuclear translocation of the Glis family of transcription factors (Gli1, Gli2, and Gli3) that subsequently activates target gene expression [6, 10–12].

Recent studies have shown that SHH signaling is abnormally activated in neuroblastoma, colorectal cancer, basal cell cancer, medulloblastoma, prostate cancer, ovarian cancer, pancreatic cancer, and other forms of cancer [10, 13]. Some studies have suggested that SHH signaling activation could contribute to carcinogenesis [14–17]. Several studies confirmed that SHH pathway activation is associated with poorly differentiated and aggressive types of GC [18–20]. Therefore, an increased understanding of SHH signaling in carcinogenesis could provide novel insights into GC treatment. Liu et al [21]. reported that autocrine SHH signaling enhanced myeloma cell proliferation and protected cells against chemotherapy-associated spontaneous and stress-induced apoptosis. Few studies have explored the mechanism of autocrine SHH signaling on cell proliferation in GC.

In this study, we performed in vitro experiments to determine the effects of autocrine SHH signaling on GC proliferation. Moreover, we examined the expression of SHH in GC tissues and adjacent non-tumor tissues. Furthermore, we evaluated the association between SHH expression and clinical features, as well as the duration of patient survival.

## Methods

### Patient samples

Ethical approval for human subjects was obtained from the Institutional Review Board of the First Affiliated Hospital of Sun Yat-Sen University (FAHSYSU), and written consent was obtained from each patient. GC paraffin-embedded tissues were obtained from Department of Pathology. Briefly, samples from 117 GC patients, who received surgical treatment at FAHSYSU between 2004 and 2005, were collected and confirmed as GC, and then made available for this study. Follow-ups were terminated until December 2013.

Fresh tumor samples from resection specimens were collected from patients with primary GC who were treated by gastric surgery without radiotherapy or chemotherapy before surgical resection at FAHSYSU in 2014 (*N* = 30). All excised tissues were frozen immediately in liquid nitrogen and then stored at -80 °C.

The use of all tissue blocks and serum samples for this study was approved by the Institutional Ethics Review Board of FAHSYSU.

### Cell culture and reagents

Human GC cell lines AGS, SGC-7901, MGC823, HGC-27 and MKN-1 were obtained from the Cell Bank of Chinese Academy of Medical Science (Shanghai, China). These cells were cultured in Dulbecco’s modified Eagle’s medium containing 10 % fetal bovine serum (Invitrogen Life Technology, Carlsbad, CA), penicilin (100 U/mL), and streptomycin (100 mg/mL). Recombinant SHH was purchased from R&D Systems, Minneapolis, MN.

### Collection of condition medium

The GC cells were grown in 15 cm petri dishes until ~80 % confluency. The medium was aspirated off, and monolayer was washed three times with PBS, once with serum-free RIMP-1640, and then replenished with serum-free RIMP-1640. After 48 h incubation, medium was collected, filtered and stored at -80 °C until use.

### Immunohistochemical (IHC) staining

For IHC, deparaffinized sections were pretreated with 10 mM sodium citrate buffer for antigen unmasking (pH 6.0, boiling temperature, 30 min), blocked in normal serum (Vectastain ABC kit, Vector Laboratories, Inc. Burlingame, CA), incubated with primary antibodies at 4oC overnight, rinsed, and incubated with secondary antibody (Vectastain ABC kit). Signals were amplified using Vectastain ABC kit per manufacturer’s instruction. Targeted protein was visualized using diaminobenzidine as substrate. The results were interpreted by two independent pathologists who were blinded to the specific diagnosis and prognosis for each case, and were scored by a semi-quantitative method in which staining of more than 10 % of the tumor cells were considered positive. The staining intensity was scored as “negative”,“weak staining”, “moderated staining” and “strong staining”. Low SHH expression was determined by negative and weak staining, and high SHH expression was determined by moderate and strong staining.

### Western blot

Total protein was extracted with cell lysis buffer and the protein concentration was quantified using an Enhanced BCA Protein Assay Kit. Protein was separated by 8–10 % SDS-PAGE and electrotransferred to PVDF membranes (Millipore, Billerica, MA, USA). The membrane was blocked for 1 h with 5 % BSA in TBS-T, and probed with corresponding primary antibodies overnight at 4uC, followed by incubation with rabbit and mouse radish peroxidase-coupled secondary antibodies for 1 h. Specific bands were detected using the enhanced chemiluminescence reagent (Millipore, Billerica, MA, USA) on autoradiographic film. The primary antibodies used were as follows: anti-SHH, SHH-neutralization antibody (Abcam, USA), anti-PLCγ1, anti-phosphorylated PLCγ1, anti-ERK1/2, anti-phosphorylated ERK1/2 (Cell Signaling Technology, Danvers, MA, USA), anti-GAPDH (Proteintech, Wuhan, China), PLCγ1 inhibitor (U73122, Sellock, Shanghai, China).

### Quantitative real-time polymerase chain reaction (qRT-PCR)

Total RNA was isolated using RNA plus reagent (TaKaRa, Japan). Complementary DNA was prepared using oligodT primers according to the protocol supplied with the Primer Script TM RT Reagent (TaKaRa, Japan). Expression of SHH was determined by quantitative real-time PCR using Power SYBR green PCR master mix (Applied Biosystems).

### Proliferation assay

Cell counting Kit-8 (CCK-8) assay was used to detect cell proliferation. In brief, cells were seeded onto 96-well cell culture cluster plates (KeyGene, Nanjing, China) at a density of 2 × 10^3^ cells/well in 100 μL culture after treating with CM in the presence or absence of SHH-NA, rhSHH and U73122for 48 h. Then, 10 μL CCK-8 reagents (Dongjido, Japan) were added to each well for 2 h incubation at 37 °C according to the manufacturer’s instructions. The absorbance was read at the wavelength of 450 nm in an automated plate reader. The experiments have been repeated at least three times.

### Enzyme-linked immunosorbent assays (ELISA)

The secreted SHH levels were detected by ELISA. One hundred microliters of cell supernatant was used for the SHH assay using an ELISA kit (Lifespan, BioSciences, USA). Briefly, a total of 100 μl per well of condition medium and standard solution were added to antibody coated 96 well plates and incubated for 2 h at room temperature, followed by addition of biotin-conjugated polyclonal antibody specific for SHH and incubation for one hour. The plate was then washed and incubated with avidin conjugated to HRP (Lifespan BioSciences, USA) for 1 h. Color was developed using TMB substrate (eBioscience), stoped by adding sulfuric acid and measured using a plate reader (M200 Pro, Tecan) at a wavelength of 450 nm.

### Statistical analyses

The SPSS ver 18.0 (SPSS Inc, Chicago, IL) was used for analysis of the data. The relationship between SHH expression and features of tumor progression were analyzed using the Chi-square and the Fisher’s exact tests. Kaplan–Meier survival curves were constructed and the log-rank test was carried out in univariate analysis. Multivariate analysis was performed using Cox’s proportional hazards model. A *P*-value of 0.05 was considered to be statistically significant for all analyses.

## Results

### Increased levels of SHH in peripheral blood and tumor tissue of GC patients

**Fig. 1:**
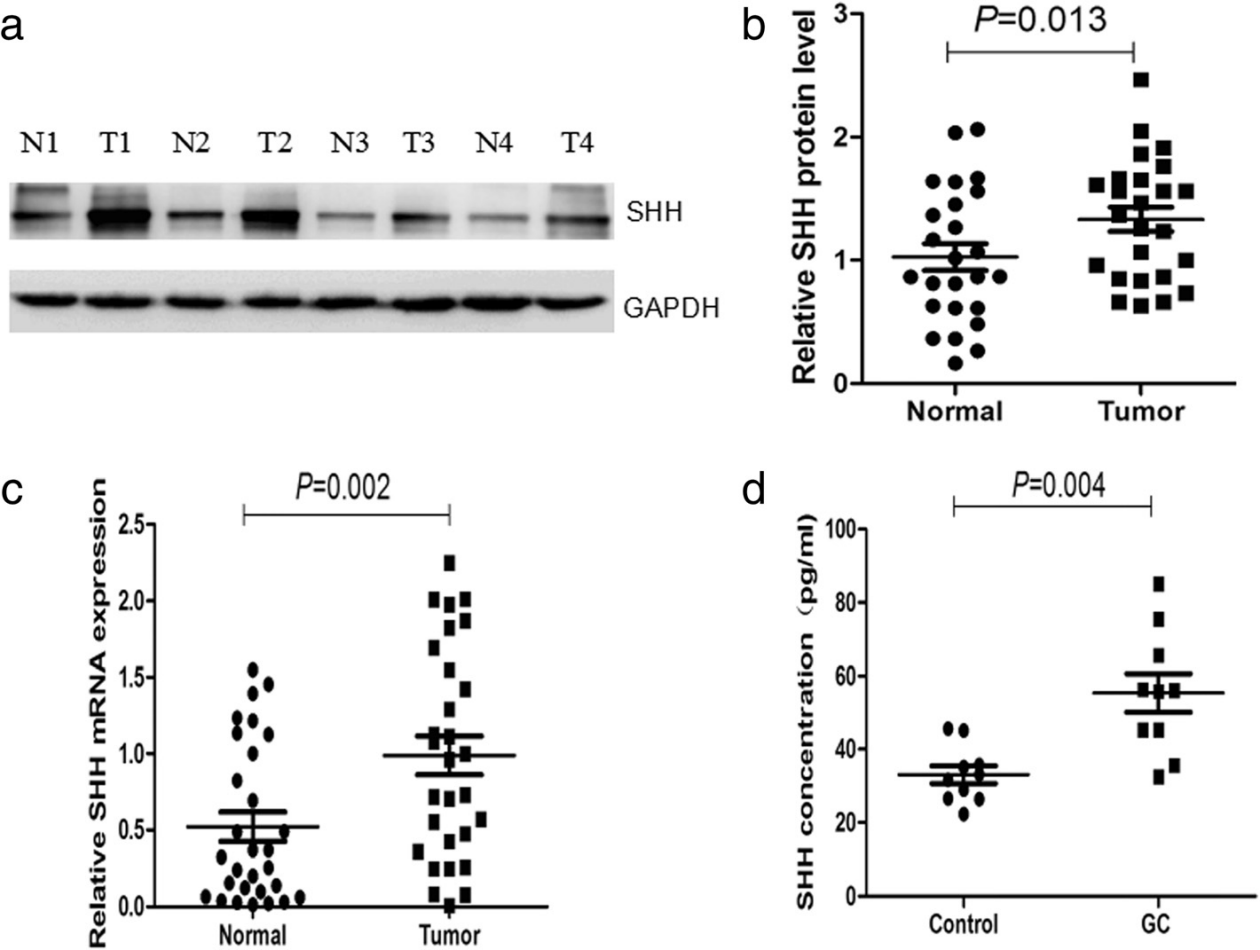
SHH protein and mRNA levels are increased in GC tissue. **a** SHH protein expression was analyzed using western blot in tumor (T) and normal (N) gastric tissues in 4 typical samples. **b** Scatter plots of the densitometrical data show the distribution of SHH expression in tumor and normal gastric tissues (*n* = 30). **c** SHH gene expression was analyzed using qRT-PCR in tumor and normal gastric tissues (*n* = 30). **d** Serum SHH was analyzed using ELISA in GC patients (*n* = 10) and control subjects (*n* = 10)

SHH protein expression in GC tissue and adjacent non-tumor tissue was analyzed using western blot in a cohort of 30 patients. We found that compared with tumor tissues, 9 patients (9/30) have low SHH expression in tumor tissues, and 21 patients (21/30) have high SHH expression in tumor tissues. SHH protein expression was significantly higher in tumor tissue compared with that in non-tumor tissue (Fig. 1a & b) (*P* = 0.013). SHH expression at the mRNA level in GC tissue and adjacent non-tumor tissue was analyzed using qRT-PCR in a cohort of ten patients. SHH gene expression was significantly higher in most tumor tissues (Fig. 1c) (*P* = 0.002). SHH mRNA expression was normalized to that of GAPDH mRNA, which served as a control for the input cDNA.

The increased SHH expression observed in GC tissue prompted us to evaluate SHH levels in GC patient blood. We quantified serum SHH concentrations in samples from 10 GC patients and 10 age-matched controls using ELISA. Serum SHH levels were higher in GC patients compared with those of age-matched health controls (Fig. 1d) (*P* = 0.004).

### Association between SHH protein expression and clinicopathologic factors in GC patients

**Fig. 2:**
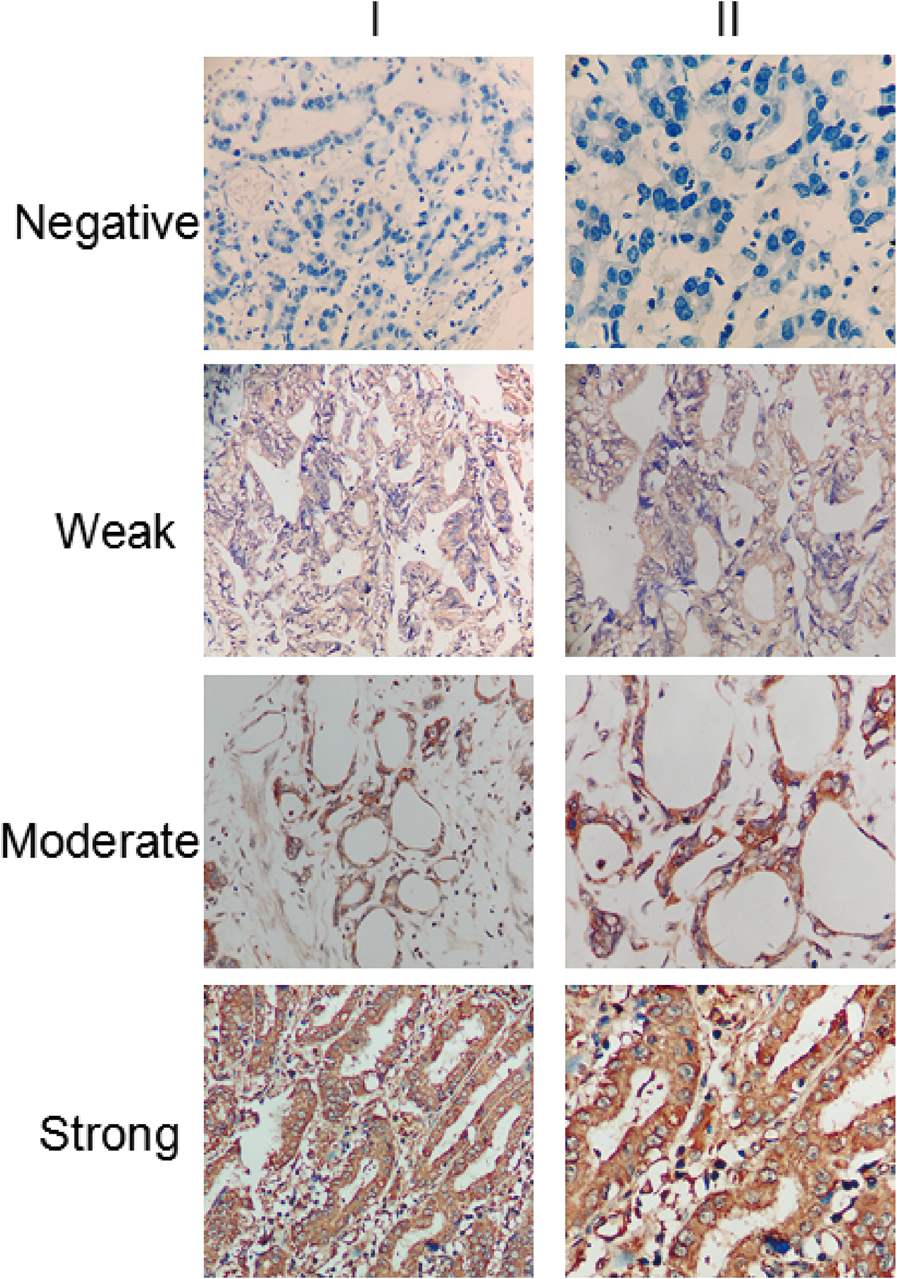
IHC staining of SHH protein in GC tissue. SHH protein expression was evaluated in GC tissue samples and classified as negative, weak, moderate, or strong. Magnification: ×200 (I) and × 400 (II)

SHH potentially contributes to GC progression, and increased SHH expression could be associated with more advanced stages of the disease. Immunohistochemistry was performed to evaluate SHH protein expression in 117 GC tissue samples (Fig. 2). Weak staining (score: 1+) was observed in 46.15 % of patients (54/117), moderate staining (score: 2+) was observed in 30.77 % of patients (36/117), strong staining (score: 3+) was observed in 10.26 % of patients (12/117) and negative staining (score: 0) was observed in 12.82 % of patients (15/117). Furthermore, as shown in Table 1, a chi-square test suggested that high SHH expression (scores of 2+ and 3+) in GC tissue samples significantly correlated with advanced distant metastasis (pM staging, 20.8 % vs 4.3 %, *P* = 0.006), and advanced TNM staging (89.6 % vs 55.1 %, *P* < 0.001). Interestingly, low expression of SHH was significantly associated with advanced tumor invasion (pT staging, 85.5 % vs 62.5 %, *P* = 0.004), increased lymph node metastasis (pN staging, 89.9 % vs 72.9 %, *P* = 0.017). However, there were no statistically significant relationships between SHH expression and other clinicopathological variables such as age (*P* = 0.479), gender (*P* = 0.444), tumor location (*P* = 0.578), tumor size (*P* = 0.223), histological type (*P* = 0.357), degree of differentiation (*P* = 0.232), and Bormann classification (*P* = 0.924).

**Table 1:** Relationship between SHH expression and clinicopathologic characteristics in GC patients

Characteristic	*N*	SHH Expression			
		Low(*n* = 69)	High(*n* = 48)	*Χ* ^2^ Value	*P* value
Gender					
Male	71	41 (59.42 %)	30 (62.50 %)	0.113	0.444
Female	46	28 (40.58 %)	18 (37.50 %)		
Age (years)					
<60 y	74	43 (62.38 %)	31 (64.58 %)	0.062	0.479
≥ 60 y	43	26 (37.62 %)	17 (35.42 %)		
Location					
Proximal	14	10 (14.49 %)	4 (8.33 %)	1.973	0.578
Middle	18	10 (14.49 %)	8 (16.67 %)		
Distal	59	32 (46.38 %)	27 (56.25 %)		
More than 2	26	17 (24.64 %)	9 (18.75 %)		
Tumor size					
<5 cm	67	37 (53.62 %)	30 (62.50 %)	0.911	0.223
≥ 5 cm	50	32 (46.38 %)	18 (37.50 %)		
Histologic type					
Adenocarcinoma	98	59 (85.51 %)	39 (81.25 %)	0.377	0.357
Others	19	10 (14.49 %)	9 (18.75 %)		
Bormann classification					
1	6	4 (5.80 %)	2 (4.17 %)	0.476	0.924
2	24	15 (21.74 %)	9 (18.75 %)		
3	69	39 (56.52 %)	30 (62.50 %)		
4	18	11 (15.94 %)	7 (14.58 %)		
Differentiation grade					
Well	26	19 (27.54 %)	7 (14.58 %)	2.924	0.232
Moderately	66	48 (69.56 %)	40 (83.33 %)		
Poorly	25	2 (2.90 %)	1 (2.08 %)		
pT staging					
T1–2	28	10 (14.49 %)	18 (37.50 %)	8.231	0.004
T3–4	89	59 (85.51 %)	30 (62.50 %)		
pN staging					
N0	20	7 (10.14 %)	13 (27.08 %)	5.731	0.017
N1-3	97	62 (89.86 %)	35 (72.92 %)		
pM staging					
M0	104	66 (95.65 %)	38 (79.17 %)	7.789	0.006
M1	13	3 (4.35 %)	10 (20.83 %)		
pTNM staging					
I–II	36	31 (44.93 %)	5 (10.42 %)	15.827	<0.001
III–IV	81	38 (55.07 %)	43 (89.58 %)		

### Correlation between SHH protein expression and the prognosis of GC patients

**Fig. 3:**
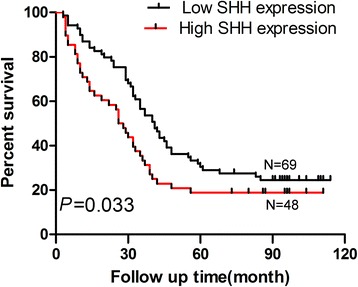
High SHH protein expression correlates with poor GC prognosis. Patients with higher SHH expression displayed a lower survival rate compared with that of patients with lower expression (*P* = 0.033)

We next evaluated the relationship between SHH expression and GC prognosis. For all patients in the study, the follow-up period ranged from 3 to 114 months, with a mean survival time of 47.6 (47.571 ± 3.590) months and a 5-year overall survival (OS) rate of 25.64 %. We used a Kaplan-Meier plot to compare survival between patient groups with low SHH expression (*N* = 69) and high SHH expression (*N* = 48). High SHH tumor expression was associated with a poor prognosis (Fig. 3, *P* = 0.033). Median survival time was 53.5 (53.517 ± 4.602) months in the low SHH expression group and 38.5 (38.542 ± 5.354) months in the high SHH expression group. The 5-year OS rate was 30.43 % in the low SHH expression group and 16.67 % in the high SHH expression group.

As shown in Table 2, a univariate analysis showed that gender (HR = 0.580, 95 % CI, 0.360–0.934, *P* = 0.025), age (HR = 1.682, 95 % CI, 1.071–2.641, *P* = 0.024), differentiation degree (HR = 0.623, 95 % CI, 0.389–0.997, *P* = 0.049), pN staging (HR = 1.652, 95 % CI, 1.221–2.234, *P* = 0.001), pM staging (HR = 3.017, 95 % CI, 1.536–5.926, *P* = 0.001), and SHH expression (HR = 1.776, 95 % CI, 1.119–2.820, *P* = 0.015) were significantly associated with GC prognosis. Furthermore, a multivariate analysis demonstrated that SHH expression status was an independent prognostic predictor in GC patients (HR = 1.734, 95 % CI, 1.109–2.713, *P* = 0.016).

**Table 2:** Univariate and multivariate analyses of clinicopathological factors for overall survival in GC patients

Variables	Univariate analysis				Multivariate analysis			
	HR	95 % CI		*P* value	HR	95 % CI		*P* value
		Lower	Upper			Lower	Upper	
Gender	0.580	0.360	0.934	0.025	0.576	0.371	0.895	0.014
Age	1.682	1.071	2.641	0.024	1.634	1.066	2.505	0.024
Tumor location	0.972	0.736	1.285	0.844				
Tumor size	1.045	0.636	1.717	0.862				
Histologic type	0.890	0.686	1.155	0.380				
Bormann classification	1.089	0.811	1.461	0.571				
Differentiation grade	0.623	0.389	0.997	0.049	0.602	0.377	0.962	0.034
pT staging	0.742	0.274	2.007	0.557				
pN staging	1.652	1.221	2.234	0.001	1.682	1.365	2.072	<0.001
pM staging	3.017	1.536	5.926	0.001	3.003	1.582	5.701	0.001
pTNM staging	1.272	0.488	3.315	0.622				
SHH expression	1.776	1.119	2.820	0.015	1.734	1.109	2.713	0.016

### SHH expression in GC cell lines

**Fig. 4:**
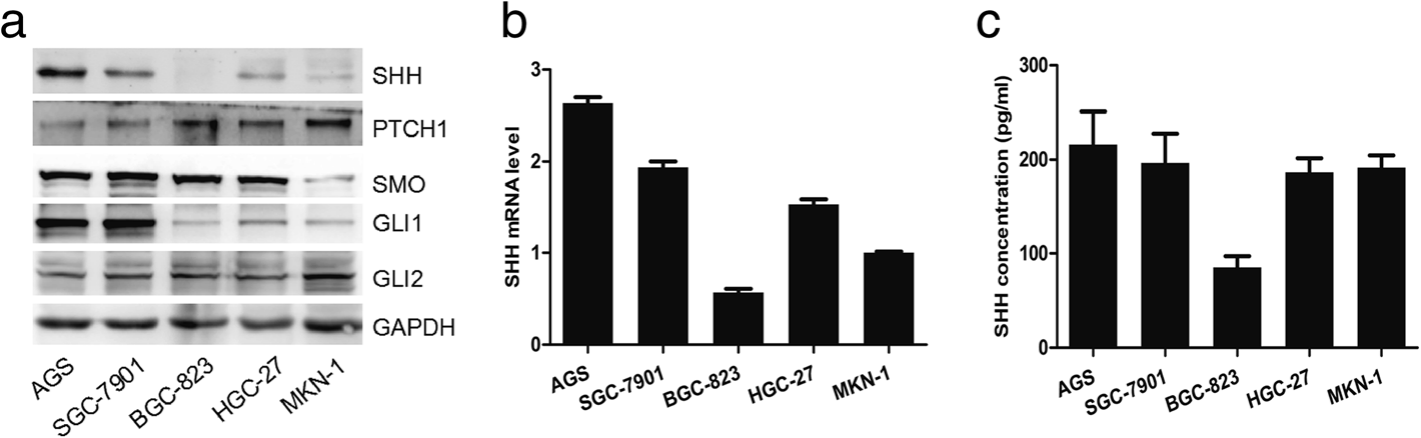
SHH expression in GC cell lines. **a** Expression of Hedgehog signaling pathway-related proteins was analyzed using western blot in GC cell lines. **b** SHH gene expression was analyzed using qRT-PCR. **c** SHH secretion was analyzed using ELISA

We determined that GC patients have a higher SHH concentration in the blood compared with that of controls. Therefore, we hypothesized that autocrine SHH signaling is required for gastric carcinogenesis. In this regard, we examined the expression of SHH signaling pathway related factors (SHH, PTCH1, SMO, GLI1) in 5 GC cell lines, including AGS, SGC-7901, BGC-823, HGC-27 and MKN-1 using western blot and qRT-PCR. By Western blot and qRT-PCR, we found that SHH is differentially expressed, with the highest level in AGS, medium level in SGC-7901 and HGC-27, and the lowest level in BGC-823 and MKN-1 (Fig. 4a & b). We also evaluated SHH concentration in conditioned media (CM) using ELISA (Fig. 4c). Based on initial analyses, we selected AGS and SGC-7901 for further experiments, as these displayed activated SHH signaling.

### Autocrine SHH signaling promotes GC cell proliferation

**Fig. 5:**
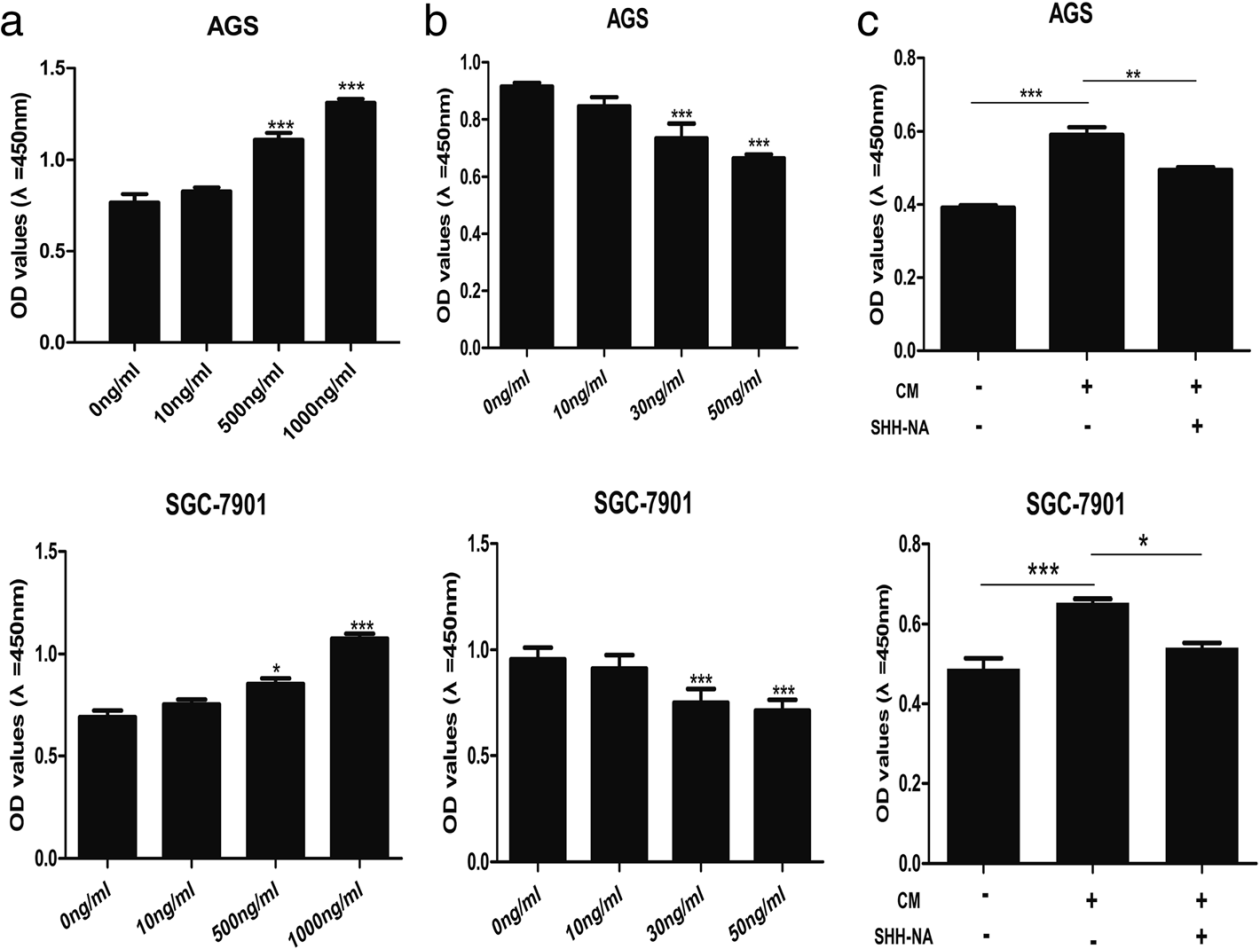
Autocrine SHH signaling affects cell proliferation in cultured GC cells. **a** Effect of Recombination Human SHH (rhSHH) treatment on proliferation of AGS (top panel) and SGC-7901 (bottom panel) cells. **b** GC cell proliferation in response to SHH-neutralizing antibody (SHH-NA) in AGS (top panel) and SGC-7901 (bottom panel). **c** AGS (top panel) and SGC-7901 (bottom panel) cell proliferation under Basic Medium (BM) and Condition Medium (CM) with or withoutSHH-NA. Mean ± SEM, *t*-test, *P < 0.05, **P < 0.01,***P < 0.001

Next, we determined whether the activity of autocrine SHH is positively associated with cell activation in GC. Cells treated with recombinant human SHH (rhSHH) displayed increased cell activity in AGS (Fig. 5a, top panel) and SGC-7901 (Fig. 5a, bottom panel) cells. Treatment with an SHH neutralizing antibody (SHH-NA) significantly decreased the proliferation of AGS (Fig. 5b, top panel) and SGC-7901 (Fig. 5b, bottom panel) cells. Specific concentrations of rhSHH (50 ng/ml) and STIP-NA (30 ng/ml) were used for further experiments.

To determine whether a similar mechanism also exists in GC, AGS and SGC-7901 cells were treated with their respective CM in the presence or absence of SHH-NA. The proliferation of AGS and SGC-7901 cells in their respective CM was significantly higher than that in basal media (BM) (Fig. 5c). SHH-NA treatment moderately decreased CM-induced cell proliferation in AGS cells (Fig. 5c, top panel) and significantly decreased proliferation in SGC-7901 cells (Fig. 5c, bottom panel), suggesting that a functional extracellular autocrine mechanism mediated by secreted SHH exists in GC cells.

### Autocrine SHH signaling promotes cell proliferation through the PLCγ1-ERK1/2 pathway

**Fig. 6:**
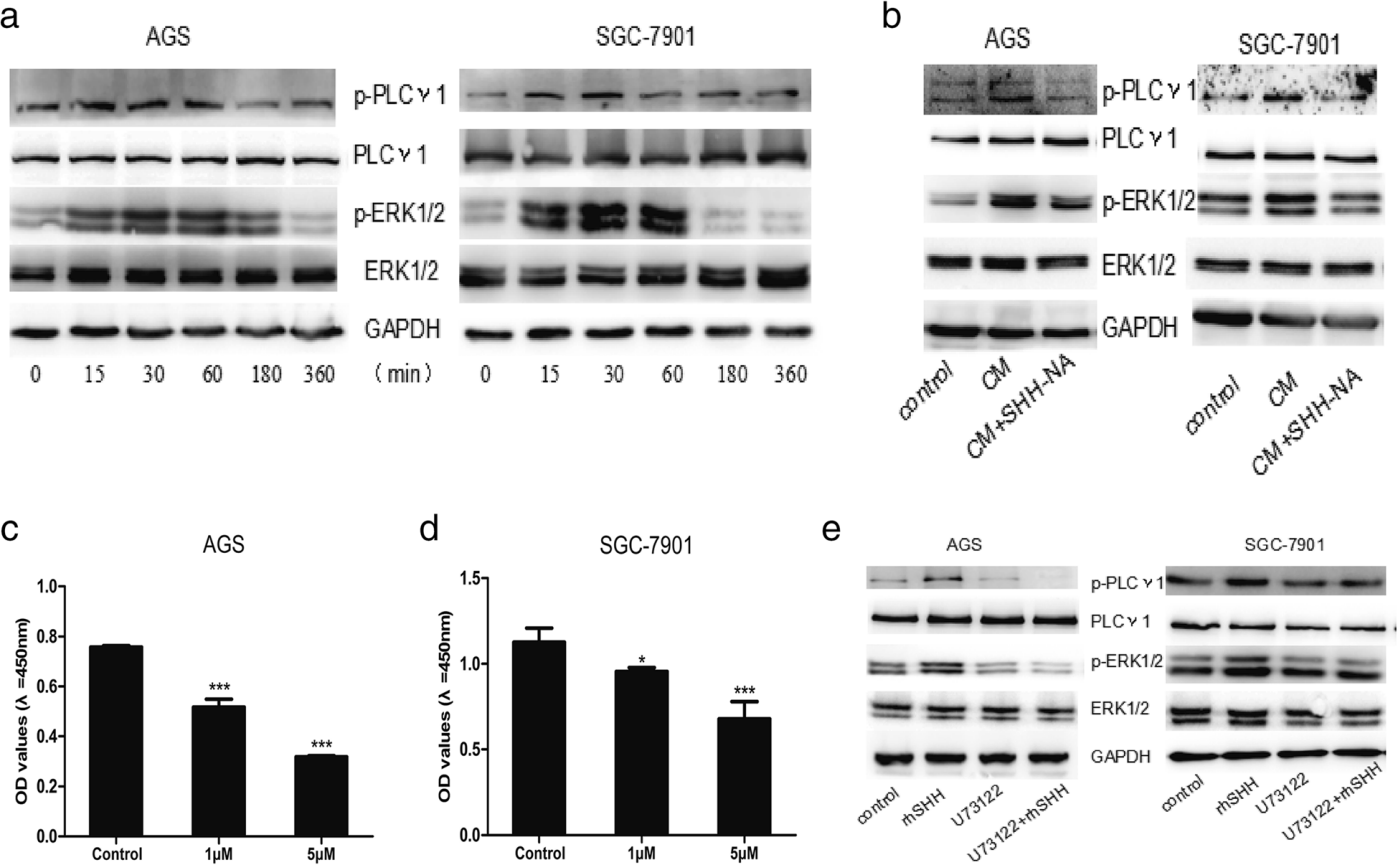
Autocrine SHH promotes cell proliferation through a PLCγ1-dependent pathway in GC cell lines. **a** Representative western blot demonstrating phosphorylation of PLCγ1 and ERK1/2 in AGS and SGC-7901 cells following Recombination Human SHH (rhSHH) treatment. **b** Western blot experiments demonstrated that Condition Medium (CM) activated phosphorylation of PLCγ1 and ERK1/2 in AGS and SGC-7901 cells, with or without SHH-neutralizing antibody (SHH-NA) treatment. **c** GC cell proliferation in response to the PLCγ1 inhibitor (U73122) in AGS cells. **d** GC cell proliferation in response to the PLCγ1 inhibitor (U73122) in SGC-7901 cells. **e** After U73122 treatment, protein levels were analyzed using western blot, with GAPDH used as a loading control. Mean ± SEM, *t*-test, *P < 0.05, **P < 0.01,***P < 0.001

To explore the underlying mechanism of autocrine SHH signaling in GC, we analyzed the downstream signaling pathways modulated by the PLCγ1-ERK1/2 pathway using western blot. We observed increased PLCγ1 and ERK1/2 phosphorylation 15 min after rhSHH treatment, which peaked between 30 and 60 min in both cell lines (Fig. 6a). These data demonstrated that autocrine SHH signaling could activated PLCγ1 and ERK1/2 in a time dependent fashion.

To determine whether the autocrine SHH-mediated cell proliferation was activated through the PLCγ1-ERK1/2 pathway, cells were treated with their respective CM in the presence or absence of SHH-NA. In both cell lines, PLCγ1 and ERK1/2 phosphorylation was increased following CM treatment. In the presence of SHH-NA, CM did not promote PLCγ1 and ERK1/2 phosphorylation (Fig. 6b). That demonstrated that physiological dose of SHH also could activate PLCγ1 and ERK1/2.

To further investigate the role of PLCγ1 in GC cell proliferation, we treated GC cells with a PLC inhibitor, U73122, to determine the effect on GC cell viability. Compared with the control group (DMF only), U73122 significantly inhibited AGS (Fig. 6c) and SGC-7901 (Fig. 6d) cell proliferation at concentrations of 1 μM and 5 μM, respectively.

We next evaluated whether the PLCγ1-ERK1/2 signaling pathway was responsible for the rhSHH-induced increase in cell proliferation. Cells were treated with DMF or 5 μM of U73122 overnight followed by a 60 min exposure to rhSHH. Again, rhSHH induced cell proliferation and the phosphorylation of PLCγ1 and ERK1/2 in both cell lines. Treatment with U73122 decreased the rhSHH-induced phosphorylation of PLCγ1 and ERK1/2 to sub-baseline levels (Fig. 6e). Collectively, these data demonstrate that autocrine SHH-mediated cell proliferation was at least partially activated through the PLCγ1-ERK1/2 pathway.

## Discussion

The functions of the SHH signaling pathway have been previously explored in various types of human tumors, including B-cell lymphoma [22], malignant pleural mesothelioma [23], medulloblastoma [24, 25], pancreatic cancer [26, 27], prostate cancer [28, 29], lung cancer [30, 31], basal cell carcinoma [13], and chronic myelogeneous leukemia [32]. However, this is the first study to explore the role of autocrine SHH signaling in GC. In the present study, SHH expression was detected in freshly frozen GC tissues, and expression of SHH mRNA and protein was higher in GC tissue compared with that in matched adjacent noncancerous tissue. Importantly, we observed that SHH concentration was significantly increased in serum samples from GC patients, supporting a potential role as a GC biomarker with diagnostic value. We demonstrated that SHH is secreted by GC cells and promotes cell proliferation in an autocrine fashion through the PLCγ1-ERK1/2 signaling pathway. In vitro, higher SHH expression was associated with several tumor progression features and poorer OS in GC.

It has been reported that SHH overexpression correlates with the clinicopathologic characteristics and prognosis of GC patients. Niu et al. [18] evaluated 113 cases of GC and found that SHH overexpression correlated with age, degree of tumor differentiation, T staging, and N staging. Furthermore, SHH overexpression did not significantly correlate with OS and DFS. However, Kim et al. [19] found that patients at a lower disease stage showed higher SHH expression, and SHH overexpression was associated with a favorable prognosis in GC patients. Interestingly, Yoo et al. [20] found that SHH expression positively correlated with lymphatic metastasis and poor prognosis. Interestingly, in this study,, we observed that higher expression of SHH was significantly associated with advanced distant metastasis, and advanced TNM staging. Lower expression of SHH was significantly associated with advanced tumor invasion, increased lymph node metastasis. Survival analysis demonstrated that patients with high SHH expression have a shorter survival time compared with that of patients with low expression, suggesting that SHH expression is an independent predictor of poor survival in GC patients.

Lian and colleagues firstly proposed the paracirne manner of SHH signaling in stromal cells in prostate cancer, and demonstrated that paracrine SHH signaling could promote tumor growth [14]. Several studies also comfired that parocrine SHH signaling affects the development and differentiation of thymocyte and other cell types [33–35]. Conversely, Rhim et al. reported that inhibition of SHH siganling accelerated tumor progression in a mouse model of pancreatic adenocarcinoma, and demonstrated that paracrine SHH signaling could act to restrain, rather than promote tumor progression [36]. Additionally, the biological functions of autocrine SHH signaling have been demonstrated in morphogenesis, pathophysiologic processes, and tumorigenesis [37–39]. Liu et al [21]. reported that autocrine SHH signaling enhanced myeloma cell proliferation and protected cells against chemotherapy-associated spontaneous and stress-induced apoptosis. Similarly, in this study, we confirmed that SHH protein could be secreted outoff GC cells and into blood, and also autocrine SHH signaling could promote cell proliferation.

Activation of SHH signaling correlates with that of the insulin growth factor (IGF), PI3K-AKT, WNT, and Notch pathways [40, 41]. SHH has been shown to regulate cell proliferation and differentiation through the MAPK-ERK and PI3K-AKT signaling pathways [42]. In addition, MAPK activity was shown to play an important role in modulating SHH-mediated gene transcription in astrocytes [43]. Ge et al. [44] demonstrated that SHH signaling contributes to PFKFB3 activation via Smo and p38 MAPK/MK2, causing accelerated glycolysis and cell proliferation in breast cancer cells. We determined that treating of cells with rhSHH and CM induced PLCγ1 and ERK1/2 phosphorylation. After treating cells with U73122, an inhibitor of PLCγ1, rhSHH and CM did not activate PLCγ1 and ERK1/2. These results strongly suggest that autocrine SHH signaling promotes cell proliferation through the PLCγ1- ERK1/2 signaling pathways.

## Conclusions

In conclusion, the present study demonstrated that SHH overexpression is associated with poor GC survival. SHH expression status could represent a useful prognostic indicator and a potential therapeutic target for individualized treatment. GC tissues secrete SHH into the tumor microenvironment and eventually into the systemic circulation. Secreted SHH stimulates cell proliferation via the PLCγ1- ERK1/2 signaling pathway in an autocrine fashion. Targeting SHH could provide a novel therapeutic strategy for GC treatment, and SHH could represent a novel GC biomarker.
